# Comparison between Deep-Learning-Based Ultra-Wide-Field Fundus Imaging and True-Colour Confocal Scanning for Diagnosing Glaucoma

**DOI:** 10.3390/jcm11113168

**Published:** 2022-06-02

**Authors:** Younji Shin, Hyunsoo Cho, Yong Un Shin, Mincheol Seong, Jun Won Choi, Won June Lee

**Affiliations:** 1 Department of Electrical Engineering, Hanyang University, Seoul 04763, Korea; yjshin@spa.hanyang.ac.kr; 2 Department of Ophthalmology, Hanyang University College of Medicine, Seoul 04763, Korea; patentrookie@naver.com (H.C.); yushin@hanyang.ac.kr (Y.U.S.); goddns76@hanmail.net (M.S.)

**Keywords:** deep learning, image processing, glaucoma, diagnostic ability

## Abstract

In this retrospective, comparative study, we evaluated and compared the performance of two confocal imaging modalities in detecting glaucoma based on a deep learning (DL) classifier: ultra-wide-field (UWF) fundus imaging and true-colour confocal scanning. A total of 777 eyes, including 273 normal control eyes and 504 glaucomatous eyes, were tested. A convolutional neural network was used for each true-colour confocal scan (Eidon AF™, CenterVue, Padova, Italy) and UWF fundus image (Optomap™, Optos PLC, Dunfermline, UK) to detect glaucoma. The diagnostic model was trained using 545 training and 232 test images. The presence of glaucoma was determined, and the accuracy and area under the receiver operating characteristic curve (AUC) metrics were assessed for diagnostic power comparison. DL-based UWF fundus imaging achieved an AUC of 0.904 (95% confidence interval (CI): 0.861–0.937) and accuracy of 83.62%. In contrast, DL-based true-colour confocal scanning achieved an AUC of 0.868 (95% CI: 0.824–0.912) and accuracy of 81.46%. Both DL-based confocal imaging modalities showed no significant differences in their ability to diagnose glaucoma (*p* = 0.135) and were comparable to the traditional optical coherence tomography parameter-based methods (all *p* > 0.005). Therefore, using a DL-based algorithm on true-colour confocal scanning and UWF fundus imaging, we confirmed that both confocal fundus imaging techniques had high value in diagnosing glaucoma.

## 1. Introduction

Glaucoma is a chronic, progressive optic nerve disease with characteristic visual field (VF) impairment resulting from the loss of the retinal nerve fibre layer (RNFL). The early diagnosis of the disease is difficult because the symptoms are not clear until it has progressed to the end stage. Nevertheless, early diagnosis is important for preventing visual impairment caused by glaucomatous damage. The visualisation of RNFL defects is a useful indicator for the early detection of glaucomatous damage [1,2,3,4,5]. In the very early stages of glaucoma, detecting RNFL defects is critical for diagnosing it; the RNFL has a relatively high diagnostic value because damage to it can appear before VF damage [6,7].

Several imaging devices are used to diagnose glaucoma. As many types of imaging methods can be used, physicians usually integrate the results obtained from multiple methods and perform the diagnosis based on such. Therefore, efforts to improve diagnosis using various imaging methods have been made in the fields of ophthalmology and glaucoma [8].

Recently, high-resolution fundus imaging via confocal scanning has been developed and has begun to be widely used in clinical practice. Ultra-wide-field (UWF) fundus imaging and true-colour confocal scanning are widely performed. High-resolution fundus imaging equipment uses a confocal scanning laser method. UWF fundus imaging (Optomap™, Optos PLC, Dunfermline, UK) combines data captured using red and green laser sources. It is advantageous in that it is possible to capture the data within a short time period without mydriasis and observe a wide range of peripheral retinas [9,10]. True-colour confocal scanning (Eidon AF™, CenterVue, Padova, Italy) allows the emission of light from a white light-emitting diode (LED) at a particular optical wavelength to acquire a high-resolution image of 14 megapixels [11]. It can also be used to capture data within a short time period without mydriasis. These two confocal fundus imaging modalities have been introduced relatively recently and are widely used for health screening purposes in medical check-up centres as well as for glaucoma diagnosis in ophthalmology clinics. In UWF fundus imaging, red-free fundus images can be obtained by only using one laser source and a blue-reflectance image, a part of which is similar to the conventional red-free fundus images obtained using a red-free filter. In true-colour confocal scanning, red-free images can be obtained by only capturing the image of a specific wavelength region using a software that processes the image obtained using an LED light source [12].

Many efforts have been made to incorporate deep learning (DL) into various imaging methods for diagnosing ophthalmologic diseases [13,14,15,16,17,18,19,20,21,22,23,24,25]. There are many reports on the application of DL methods to conventional fundus imaging; [16,26] recently, it was reported that when optical coherence tomography (OCT) and DL methods are combined, excellent glaucoma diagnostic results can be achieved [27,28,29].

Some studies have shown that UWF fundus imaging is useful for diagnosing glaucoma [30], whereas others have shown that accurate glaucoma diagnosis can be achieved by combining UWF fundus imaging with DL methods [31,32,33,34]. However, no studies have shown the results of true-colour confocal scanning in the field of glaucoma, and this modality has only been used for the diagnosis of diabetic retinopathy and retinal diseases [35,36,37].

In a previous study conducted by our team, true-colour confocal scanning was found to be superior to UWF fundus imaging in detecting localised RNFL defects (early changes in glaucoma) when eye physicians manually evaluated images taken using both modalities [12,30,31]. It is widely known that a high false-positive rate is obtained when UWF fundus imaging is used to manually determine localised RNFL defects. Therefore, by applying DL to true-colour confocal scanning and UWF fundus imaging, we compared the diagnostic power of these test methods to determine the kind of change that occurs therein. Furthermore, we evaluated the diagnostic power of both relatively recent confocal fundus imaging techniques using a DL-based algorithm and compared them with conventional OCT parameter-based methods in terms of glaucoma diagnosis.

## 2. Methods

### 2.1. Participants

The study protocol was approved by the institutional review board of Hanyang University Hospital. This study design followed the tenets of the Declaration of Helsinki for biomedical research.

In this retrospective, cross-sectional study, we investigated 777 eyes, which included 273 healthy eyes and 504 glaucomatous eyes. All participants visited the glaucoma clinic of Hanyang University Hospital between August 2019 and August 2021. All of them underwent an ophthalmologic examination, including a visual acuity test, slit-lamp examination, intraocular pressure (IOP) measurement using Goldmann applanation tonometry, gonioscopy, axial length measurement (IOLMaster; Carl Zeiss Meditec, Dublin, CA, USA), stereo disc imaging, red-free RNFL imaging (Eidon AF™), Swedish interactive thresholding algorithm 24-2 perimetry (Humphrey Field Analyzer II; Carl Zeiss Meditec, Jena, Germany), and swept-source OCT (DRI-OCT Triton; Topcon, Tokyo, Japan).

The definition of a normal VF, glaucomatous VF defects, and the reliability of the VF test was determined according to the classical method used in many previous studies in the literature [38].

Patients with best-corrected visual acuity ≥ 20/40, refractive errors within a ±6.0 D spherical equivalent, and a ±3.0 D astigmatism were included in this study. Exclusion criteria were a history of surgical therapy (e.g., glaucoma-filtering surgery; however, patients who only underwent cataract surgery were not excluded), any other ocular disease that could affect visual function, any media opacity that would significantly hinder OCT image acquisition, and an inability to acquire high-quality images (i.e., image quality scores of <50). When both eyes met all the eligibility criteria, one eye was randomly selected for inclusion [27].

Patients with glaucoma were identified on the basis of several clinical signs. Specifically, the presence of a characteristic optic disc (i.e., neuroretinal rim thinning, notching, excavation, or a cup-to-disc ratio difference of >0.2 between the eyes) on stereo disc images was used as a major clinical sign for glaucoma diagnosis. The presence of RNFL defects on red-free fundus imaging is an alternative sign, regardless of the presence or absence of glaucomatous VF defects [27].

Healthy patients were defined as satisfying all of the following criteria: (1) patients with no history of intraocular surgery, (2) IOP of ≤21 mmHg with no history of increased IOP, (3) no glaucomatous disc appearance, and (4) normal ophthalmologic findings. Two glaucoma specialists (M.S. and W.J.L.), who were masked to all other patient information, independently judged the images. In order to avoid ambiguity, cases in which the two examiners concluded differently were excluded from the training and test datasets [27].

### 2.2. Glaucoma Diagnosis Using UWF Fundus Imaging and True-Colour Confocal Scanning

**Figure 1:**
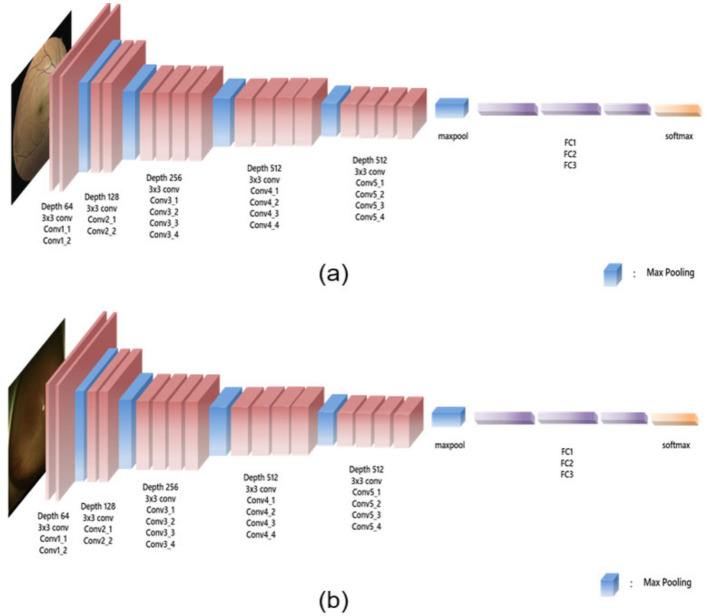
VGG-19 network. The proposed architecture used true-colour confocal scanning (**a**) and ultra-wide-field fundus (**b**) images as the input images. The VGG-19 network consisted of 16 convolutional layers and 5 pooling layers, and only a 3(×)3 kernel was used. Subsequently, a fully connected layer and softmax were applied to obtain the final output.

Convolutional neural networks (CNNs) are widely used in image-recognition tasks. They effectively extract spatial features by conducting two-dimensional convolution with kernels whose weights are determined by training the model with the dataset. Herein, a CNN was applied to detect glaucoma using true-colour confocal scanning and UWF fundus images separately. Among the various CNN architectures, the VGG-19 network was selected owing to its popularity and computational efficiency. The VGG-19 architecture is illustrated in Figure 1.

The VGG-19 network consisted of 16 convolution layers and 5 pooling layers. The last layer consisted of the fully connected layer, followed by the softmax function that produced the probability values for each category. It was pretrained with ImageNet. Using the collected dataset, we trained the VGG-19 network to perform a glaucoma diagnosis task. A batch size of 4, a learning rate of 0.001, and an epoch of 40 were used on the basis of empirical parameter tuning.

### 2.3. Statistical Analyses

To evaluate the diagnostic ability for glaucoma, we calculated the area under the receiver operating characteristic curve (AUC) and accuracy. The cut-off value for glaucoma probability was changed using the AUC of the 95% CI, and the accuracy was used as a measure of precision when classifying the stages of glaucoma. The precision–recall curve is a metric for evaluating the model of the classifier and is used when the distribution of data labels is uneven. The x-axis represents the recall, and the y-axis represents the precision. The area value is used to evaluate the performance of the binary classifier.

*p*-values of <0.05 were considered significant. Values were expressed as means ± standard deviations. Statistical tests were performed using SPSS v24 (IBM Inc., Armonk, NY, USA), MedCalc (MedCalc Software, Ostend, Belgium), and the PyTorch framework using Python (Facebook AI Research Laboratory, Menlo Park, CA, USA) [39].

## 3. Results

We split the entire dataset into training and test datasets at a ratio of 7:3. The demographic and clinical characteristics of the participants are summarised in Table 1.

### Healthy Group versus Glaucoma Group

As shown in Table 2, the accuracy of UWF fundus imaging based on DL was 83.62%, and that of true-colour confocal scanning was 81.46%. We also evaluated the AUC of the two imaging methods. In the AUC (95% CI) analysis shown in Table 2, the DL-based method achieved an AUC of 0.904 (0.861–0.937) for UWF fundus imaging and 0.868 (0.824–0.912) for true-colour confocal scanning.

**Figure 2:**
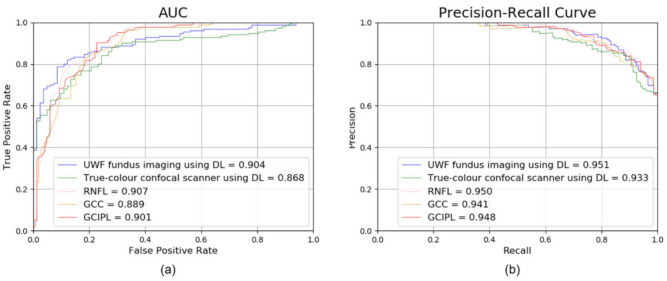
AUC (**a**) and precision–recall curves (**b**) were used for performance validation. For both the AUC and precision–recall curves, the results of the DL-based methods were similar to or higher than the results of the existing RNFL-, GCC-, and GCIPL-based methods. A dotted black line is a state where AUC is 0.5. AUC, area under the receiver operating characteristic curve; RNFL, retinal nerve fibre layer; GCIPL, ganglion cell–inner plexiform layer; GCC, ganglion cell complex.

In addition, as shown in the AUC and precision–recall curve in Figure 2, the glaucoma detection performance of the methods based on DL was similar to or better than that of the existing methods based on the RNFL, ganglion cell complex (GCC), or ganglion cell–inner plexiform layer (GCIPL).

Table 3 shows that the *p*-value between the two confocal imaging modalities was 0.135, thereby indicating no significant difference. The analysis shows that the AUC of the DL-based imaging methods did not significantly differ from that of the traditional OCT parameter-based methods (RNFL, GCIPL, and GCC, all *p* > 0.05).

## 4. Discussion

In this study, we investigated the performance of DL-based UWF fundus imaging and true-colour confocal scanning in diagnosing glaucoma. We applied DL algorithms to these two relatively recent confocal fundus imaging modalities and found that they exhibited good glaucoma diagnostic abilities compared to those of conventional OCT parameter-based methods.

Although DL has been used for diagnosing glaucoma in several studies [18,19,20,21,22,23,24,25,27,28,29,30,31], only a few have applied DL to UWF fundus images [31]. To the best of our knowledge, our study is the first to apply DL to true-colour confocal scanning images for diagnosing glaucoma. We also compared UWF fundus imaging and true-colour confocal scanning, which have not been extensively investigated in previous studies. Although a previous study compared these two imaging modalities, the images were evaluated by glaucoma specialists and not based on DL, as was performed in our study [12]. In a previous study, a large amount of UWF fundus imaging data was used to diagnose glaucoma using DL, and a high diagnostic accuracy was reported [31]. The diagnostic ability reported in that study (AUC = 0.983) was higher than that in our study (DL-based UWF fundus imaging: AUC = 0.904). This might be attributed to the fact that the previous study used a larger amount of data than that used in this study. If various methods are applied to overcome the limited data size, for example, using data augmentation, in a future study, a higher detection accuracy could be achieved with our method.

Because of the high resolution of the images, true-colour confocal scanning has been used to evaluate some retinal diseases. Some studies used true-colour confocal scanning using the EyeArt software to diagnose diabetic retinopathy [36,37]. These previously published studies that performed true-colour confocal scanning investigated the detection of retinal disease rather than glaucoma and used the EyeArt software rather than a DL algorithm. To the best of our knowledge, our study is the first to use DL-based true-colour confocal scanning to diagnose glaucoma.

Herein, we used VGGNet among the various available architectures for imaging analysis (AlexNet, VGGNet, and ResNet) [40,41,42]. VGGNet has a larger capacity than AlexNet [40,41]. Although ResNet has a stronger expression capability than VGGNet, it can easily overfit if the amount of training data is not sufficient to train the model [42]. Therefore, VGGNet was considered a reasonable option for this study. For confirmation, the performance data obtained using AlexNet and ResNet are also provided in Table S1.

We compared the diagnostic ability of the methods based on DL with that of conventional methods based on the peripapillary RNFL, macular GCIPL, and macular GCC thickness values. The conventional methods employ widely used OCT parameters for diagnosing glaucoma. Our analyses showed that the diagnostic ability of the DL-based methods was not significantly different from that of existing OCT parameter-based methods. Our study suggests that the accuracy of glaucoma diagnosis is comparable between OCT parameter- and DL-based methods.

Many studies have applied DL to fundus images [11,13,26,30,31,32,34,35]; however, our work is different from existing studies in that DL was designed to be applied to the two new confocal fundus imaging techniques with high resolution, and the diagnostic power of the two methods was compared. We believe that our analysis will be useful to the community, because these two methods are often used for screening in health check-up centres and primary clinics.

Our team compared UWF fundus imaging and true-colour confocal scanning for detecting localised RNFL defects in a previous study [12], in which the two types of images were evaluated by glaucoma specialists, not based on DL. A previous study has shown that true-colour confocal scanning could have advantages over UWF fundus imaging in evaluating localised RNFL defects [12]. UWF fundus imaging showed a significantly lower specificity than true-colour confocal scanning, which indicates that UWF fundus imaging yields a high false-positive rate. One study by another group that compared the detection of RNFL defects between conventional red-free fundus imaging and UWF fundus imaging also reported that UWF fundus imaging showed a comparable sensitivity but a lower specificity than conventional imaging [30].

Unlike the comparison made by glaucoma specialists [12], our analysis showed no significant difference in glaucoma diagnosis between the two modalities, and both yielded sufficiently good results when we adopted the DL algorithm.

The technical differences between UWF fundus imaging and true-colour confocal scanning are as follows: (1) differences in light source: a two-wavelength laser light versus an LED and a difference between false colour and true colour; (2) the confocal aperture of UWF fundus imaging is circular, whereas true-colour confocal scanning uses a confocal slit; and (3) there is a difference in the distortion owing to the different capture ranges of the two devices used.

These factors can cause differences in the interpretation of images by physicians (humans). These are possible disadvantages of UWF fundus imaging when DL is used to detect glaucoma instead of physician observations. Interestingly, the relatively distorted UWF fundus image showed a comparable diagnostic performance to the true-colour confocal scanning image (after adopting AI). Imaging not only the optic nerve but also RNFL defects is very important in diagnosing glaucoma, although the primary origin of glaucomatous damage is the optic nerve.

This study has several limitations. First, a direct comparison between the DL algorithm and judgement by physicians was not performed. Instead, we compared DL with OCT parameters that are often used. Second, because conventional fundus images or red-free images were not available, a direct comparison was difficult. When physicians discriminate against glaucoma and set the gold standard for comparison, it would be a major disadvantage to use a true-colour confocal scanning image instead of a red-free image. However, true-colour confocal scanning images have been used for the diagnosis of glaucoma in many previous studies instead of red-free images [27,43,44], and glaucoma specialists have set the gold standard (glaucoma or normal) using several other tests (stereo disc imaging or the VF test). It is expected that this limitation can be overcome to some extent because the diagnostic decisions were made by specialists based on images using the gold standard, and all other analyses were only performed via artificial intelligence. Third, age differences were observed between the glaucoma and normal groups. A large amount of data was collected in actual clinical settings; therefore, the high prevalence of glaucoma in elderly populations could affect our results. However, because the analysis only used images based on DL and no difference was found between the training and test datasets, it did not have a significant effect on the main results of this study. Finally, to check the results of the limited dataset (small sample size), we performed cross-validation using different samples for training by dividing the images into five test groups and evaluating the accuracy for each group. Tables S2 and S3 show the accuracy obtained for each test group.

## 5. Conclusions

Our study results confirmed that the ability of DL-based UWF fundus imaging and true-colour confocal scanning to diagnose glaucoma was comparable to that of OCT parameter-based methods. By adopting DL, these two recently developed confocal fundus imaging systems are expected to be useful for glaucoma diagnosis.

## Figures and Tables

**Table 1:** Demographic and clinical characteristics of the eyes and patients in the training versus test samples.

	Overall			Training			Test			*p*-Value
	Overall	Normal	Glaucoma	Overall	Normal	Glaucoma	Overall	Normal	Glaucoma	
N	777	274 (35.3%)	503 (64.7%)	545	192 (35.2%)	353 (64.8%)	232	82 (35.3%)	150 (64.7%)	0.975
Sex (M)	417 (53.7%)	142 (51.8%)	275 (54.7%)	302 (55.4%)	103 (53.6%)	199 (56.4%)	115 (49.6%)	39 (47.6%)	76 (50.7%)	0.135
Age (year)	58.4 ± 15.8	53.3 ± 16.1	61.2 ± 14.9	58.2 ± 16.0	53.2 ± 16.7	60.8 ± 15.0	59.0 ± 15.3	53.6 ± 14.9	61.9 ± 14.8	0.516
SE (dioptre)	−1.49 ± 2.66	−1.43 ± 2.77	−1.52 ± 2.61	−1.58 ± 2.73	−1.69 ± 2.96	−1.52 ± 2.61	−1.29 ± 2.48	−0.87 ± 2.19	−1.52 ± 2.61	0.172
AXL (mm)	24.74 ± 1.75	24.71 ± 1.79	24.75 ± 1.73	24.78 ± 1.84	24.71 ± 1.86	24.81 ± 1.83	24.64 ± 1.52	24.70 ± 1.59	24.62 ± 1.50	0.526
IOP (mmHg)	14.9 ± 2.9	15.0 ± 3.0	14.8 ± 2.9	15.0 ± 3.0	15.4 ± 3.4	15.1 ± 3.4	14.6 ± 2.7	14.9 ± 2.9	14.5 ± 3.3	0.131
MD (dB)	−6.0 ± 7.4	−2.1 ± 2.7	−7.7 ± 8.1	−5.8 ± 7.2	−2.1 ± 2.0	−7.3 ± 8.0	−6.6 ± 7.9	−2.0 ± 208	−8.6 ± 8.3	0.180
VFI (%)	84.9 ± 23.1	97.0 ± 5.0	79.9 ± 25.6	85.6 ± 22.4	97.3 ± 3.0	80.8 ± 24.9	83.3 ± 24.5	96.5 ± 7.7	77.7 ± 27.0	0.241
RNFL (µm)	84.6 ± 21.4	103.0 ± 12.0	74.6 ± 18.6	84.7 ± 21.7	103.2 ± 12.9	74.6 ± 18.6	84.5 ± 20.9	102.4 ± 9.8	74.7 ± 18.8	0.899
GCIPL (µm)	61.9 ± 9.7	69.9 ± 5.5	57.6 ± 8.7	61.9 ± 10.1	70.1 ± 5.9	57.4 ± 9.1	61.9 ± 8.7	69.3 ± 4.6	57.9 ± 7.7	0.983
GCC (µm)	94.9 ± 14.4	107.3 ± 7.4	88.1 ± 12.7	95.0 ± 15.0	108.1 ± 7.4	87.8 ± 13.2	94.6 ± 13.0	105.3 ± 7.1	88.8 ± 11.7	0.753

Note: SE, spherical equivalent; AXL, axial length; IOP, intraocular pressure; MD, mean deviation; VFI, visual field index; RNFL, retinal nerve fibre layer; GCIPL, ganglion cell–inner plexiform layer; GCC, ganglion cell complex. *p*-value for comparison between the training and test datasets; comparisons were performed using the chi-square test for categorical variables and the independent t-test for continuous variables.

**Table 2:** Accuracy prediction for the diagnosis of glaucoma.

	Accuracy (%)	AUC (95% CI)
UWF fundus imaging using DL	83.62	0.904 (0.861–0.937)
True-colour confocal scanner using DL	81.46	0.868 (0.824–0.912)
RNFL	84.40	0.907 (0.871–0.947)
GCIPL	80.60	0.901 (0.862–0.941)
GCC	81.45	0.889 (0.850–0.933)

UWF, ultra-wide-field; DL, deep learning; RNFL, retinal nerve fibre layer; GCIPL, ganglion cell–inner plexiform layer; GCC, ganglion cell complex; AUC, area under the receiver operating characteristic curve; CI, confidence interval.

**Table 3:** Comparison of accuracy of diagnosis glaucoma.

	UWF Fundus Imaging Using DL	True-Colour Confocal Scanner Using DL	RNFL	GCIPL	GCC
UWF fundus imaging using DL	NA	0.135	0.759	0.998	0.645
True-colour confocal scanner using DL	*0.360*	NA	0.077	0.215	0.421
RNFL	*0.003*	*0.039*	NA	0.683	0.324
GCIPL	*0.003*	*0.033*	*0.006*	NA	0.274
GCC	*0.001*	*0.021*	*0.018*	*0.012*	NA

UWF, ultra-wide-field; DL, deep learning; RNFL, retinal nerve fibre layer; GCIPL, ganglion cell–inner plexiform layer; GCC, ganglion cell complex; NA, Not Applicable. The value above the diagonal line shows the p-value for testing difference and the value below the line shows the actual difference value of the area under the receiver operating characteristic curve (italic).

## Data Availability

Data are available upon reasonable request.

## Supplementary Materials

The following supporting information can be downloaded at: https://www.mdpi.com/article/10.3390/jcm11113168/s1, Table S1: CNN accuracy; accuracy when comparing UWF fundus images and true-colour confocal scanning images through various algorithms; Table S2: K-fold cross validation verification (k = 5); k-fold cross-validation conducted to overcome the small sample size and to solve the overfitting problem in the test for UWF fundus images; Table S3: K-fold cross-validation verification (k = 5); k-fold cross-validation conducted to overcome the small sample size and to solve the overfitting problem in the test for true-colour confocal scanning images.

## References

[1] Sommer A., Katz J., Quigley H.A., Miller N.R., Robin A.L., Richter R.C., Witt K.A. (1991). Clinically detectable nerve fiber atrophy precedes the onset of glaucomatous field loss. Arch. Ophthalmol..

[2] Sommer A. (1995). Retinal Nerve Fiber Layer. Am. J. Ophthalmol..

[3] Quigley H.A., Katz J., Derick R.J., Gilbert D., Sommer A. (1992). An evaluation of optic disc and nerve fiber layer examinations in monitoring progression of early glaucoma damage. Ophthalmology.

[4] Hoyt W.F., Frisen L., Newman N.M. (1973). Fundoscopy of nerve fiber layer defects in glaucoma. Investig. Ophthalmol. Vis. Sci..

[5] Sommer A., Miller N.R., Pollack I., Maumenee A.E., George T. (1977). The nerve fiber layer in the diagnosis of glaucoma. Arch. Ophthalmol..

[6] Jonas J.B., Schiro D. (1994). Localised wedge shaped defects of the retinal nerve fibre layer in glaucoma. Br. J. Ophthalmol..

[7] Tuulonen A., Airaksinen P.J., Montagna A., Nieminen H. (1990). Screening for glaucoma with a non-mydriatic fundus camera. Acta Ophthalmol..

[8] Kim K.E., Oh S., Jeoung J.W., Suh M.H., Seo J.H., Kim M., Park K.H., Kim D.M., Kim S.H. (2016). Spectral-domain optical coherence tomography in manifest glaucoma: Its additive role in structural diagnosis. Am. J. Ophthalmol..

[9] Patel S.N., Shi A., Wibbelsman T.D., Klufas M.A. (2020). Ultra-widefield retinal imaging: An update on recent advances. Ther. Adv. Ophthalmol..

[10] Witmer M.T., Kiss S. (2013). Wide-field imaging of the retina. Surv. Ophthalmol..

[11] Borrelli E., Lei J., Balasubramanian S., Uji A., Cozzi M., Sarao V., Lanzetta P., Staurenghi G., Sadda S.R. (2018). Green emission fluorophores in eyes with atrophic age-related macular degeneration: A colour fundus autofluorescence pilot study. Br. J. Ophthalmol..

[12] Lee J.S., Seong M., Lee W.J. (2021). Comparison between Two Modalities for Diagnosis of Localized Retinal Nerve Fiber Layer Defect: Ultra-wide field fundus photography versus True-color Confocal Scanning Images. J. Korean Glaucoma Soc..

[13] Shibata N., Tanito M., Mitsuhashi K., Fujino Y., Matsuura M., Murata H., Asaoka R. (2018). Development of a deep residual learning algorithm to screen for glaucoma from fundus photography. Sci. Rep..

[14] Raghavendra U., Fujita H., Bhandary S.V., Gudigar A., Tan J.H., Acharya U.R. (2018). Deep convolution neural network for accurate diagnosis of glaucoma using digital fundus images. Inf. Sci..

[15] Kim K.E., Kim J.M., Song J.E., Kee C., Han J.C., Hyun S.H. (2020). Development and Validation of a Deep Learning System for Diagnosing Glaucoma Using Optical Coherence Tomography. J. Clin. Med..

[16] Ahn J.M., Kim S., Ahn K.-S., Cho S.-H., Lee K.B., Kim U.S. (2018). A deep learning model for the detection of both advanced and early glaucoma using fundus photography. PLoS ONE.

[17] Wang H., Hu J., Zhang J. (2021). SCRD-Net: A Deep Convolutional Neural Network Model for Glaucoma Detection in Retina Tomography. Complexity.

[18] Liu H., Li L., Wormstone I.M., Qiao C., Zhang C., Liu P., Li S., Wang H., Mou D., Pang R. (2019). Development and Validation of a Deep Learning System to Detect Glaucomatous Optic Neuropathy Using Fundus Photographs. JAMA Ophthalmol..

[19] Christopher M., Belghith A., Bowd C., Proudfoot J.A., Goldbaum M.H., Weinreb R.N., Girkin C.A., Liebmann J.M., Zangwill L.M. (2018). Performance of Deep Learning Architectures and Transfer Learning for Detecting Glaucomatous Optic Neuropathy in Fundus Photographs. Sci. Rep..

[20] Fu H., Cheng J., Xu Y., Zhang C., Wong D.W.K., Liu J., Cao X. (2018). Disc-Aware Ensemble Network for Glaucoma Screening From Fundus Image. IEEE Trans. Med. Imaging.

[21] Phene S., Dunn R.C., Hammel N., Liu Y., Krause J., Kitade N., Schaekermann M., Sayres R., Wu D.J., Bora A. (2019). Deep Learning and Glaucoma Specialists: The Relative Importance of Optic Disc Features to Predict Glaucoma Referral in Fundus Photographs. Ophthalmology.

[22] Li F., Yan L., Wang Y., Shi J., Chen H., Zhang X., Jiang M., Wu Z., Zhou K. (2020). Deep learning-based automated detection of glaucomatous optic neuropathy on color fundus photographs. Graefes Arch. Clin. Exp. Ophthalmol..

[23] Hemelings R., Elen B., Barbosa-Breda J., Lemmens S., Meire M., Pourjavan S., Vandewalle E., Van de Veire S., Blaschko M.B., De Boever P. (2020). Accurate prediction of glaucoma from colour fundus images with a convolutional neural network that relies on active and transfer learning. Acta Ophthalmol..

[24] Hemelings R., Elen B., Barbosa-Breda J., Blaschko M.B., De Boever P., Stalmans I. (2021). Deep learning on fundus images detects glaucoma beyond the optic disc. Sci. Rep..

[25] Li L., Xu M., Liu H., Li Y., Wang X., Jiang L., Wang Z., Fan X., Wang N. (2020). A Large-Scale Database and a CNN Model for Attention-Based Glaucoma Detection. IEEE Trans. Med. Imaging.

[26] Ajitha S., Akkara J.D., Judy M. (2021). Identification of glaucoma from fundus images using deep learning techniques. Indian J. Ophthalmol..

[27] Shin Y., Cho H., Jeong H.C., Seong M., Choi J.W., Lee W.J. (2021). Deep Learning-based Diagnosis of Glaucoma Using Wide-field Optical Coherence Tomography Images. J. Glaucoma.

[28] Muhammad H., Fuchs T.J., De Cuir N., De Moraes C.G., Blumberg D.M., Liebmann J.M., Ritch R., Hood D.C. (2017). Hybrid Deep Learning on Single Wide-field Optical Coherence tomography Scans Accurately Classifies Glaucoma Suspects. J. Glaucoma.

[29] Shin J., Kim S., Kim J., Park K. (2021). Visual Field Inference From Optical Coherence Tomography Using Deep Learning Algorithms: A Comparison Between Devices. Transl. Vis. Sci. Technol..

[30] Kim M.J., Lee J.H., Park J.I., Choi J.Y., Sohn J., Hwang H.S., Hwang D.D. (2021). Novel utilisation of ultrawide-field fundus photography for detecting retinal nerve fibre layer defects in glaucomatous eyes. Br. J. Ophthalmol..

[31] Li Z., Guo C., Lin D., Nie D., Zhu Y., Chen C., Zhao L., Wang J., Zhang X., Dongye M. (2021). Deep learning for automated glaucomatous optic neuropathy detection from ultra-widefield fundus images. Br. J. Ophthalmol..

[32] Ohsugi H., Tabuchi H., Enno H., Ishitobi N. (2017). Accuracy of deep learning, a machine-learning technology, using ultra–wide-field fundus ophthalmoscopy for detecting rhegmatogenous retinal detachment. Sci. Rep..

[33] Tang F., Luenam P., Ran A.R., Quadeer A.A., Raman R., Sen P., Khan R., Giridhar A., Haridas S., Iglicki M. (2021). Detection of Diabetic Retinopathy from Ultra-Widefield Scanning Laser Ophthalmoscope Images: A Multicenter Deep Learning Analysis. Ophthalmol. Retina.

[34] Antaki F., Coussa R.G., Kahwati G., Hammamji K., Sebag M., Duval R. (2021). Accuracy of automated machine learning in classifying retinal pathologies from ultra-widefield pseudocolour fundus images. Br. J. Ophthalmol..

[35] Olvera-Barrios A., Heeren T.F., Balaskas K., Chambers R., Bolter L., Tufail A., Egan C., Anderson J. (2020). Comparison of true-colour wide-field confocal scanner imaging with standard fundus photography for diabetic retinopathy screening. Br. J. Ophthalmol..

[36] Olvera-Barrios A., Heeren T.F., Balaskas K., Chambers R., Bolter L., Egan C., Tufail A., Anderson J. (2021). Diagnostic accuracy of diabetic retinopathy grading by an artificial intelligence-enabled algorithm compared with a human standard for wide-field true-colour confocal scanning and standard digital retinal images. Br. J. Ophthalmol..

[37] Sarao V., Veritti D., Lanzetta P. (2020). Automated diabetic retinopathy detection with two different retinal imaging devices using artificial intelligence: A comparison study. Graefes Arch. Clin. Exp. Ophthalmol..

[38] Anderson D., Patella V. (1999). Automated Static Perimetry.

[39] Paszke A., Gross S., Chintala S., Chanan G., Yang E., DeVito Z., Lin Z., Desmaison A., Antiga L., Lerer A. Automatic differentiation in pytorch. Proceedings of the NIPS 2017 Autodiff Workshop 2017.

[40] Simonyan K., Zisserman A. (2014). Very deep convolutional networks for large-scale image recognition. arXiv.

[41] Krizhevsky A., Sutskever I., Hinton G.E. (2012). Imagenet classification with deep convolutional neural networks. Adv. Neural Inf. Process. Syst..

[42] He K., Zhang X., Ren S., Sun J. Deep residual learning for image recognition. Proceedings of the IEEE Conference on Computer Vision and Pattern Recognition.

[43] Kim Y.J., Na K.I., Lim H.W., Seong M., Lee W.J. (2021). Combined wide-field optical coherence tomography angiography density map for high myopic glaucoma detection. Sci. Rep..

[44] Kim H., Park H.M., Jeong H.C., Moon S.Y., Cho H., Lim H.W., Seong M., Park J., Lee W.J. (2021). Wide-field optical coherence tomography deviation map for early glaucoma detection. Br. J. Ophthalmol..

