# Supplementary material for: Comparison between Deep-Learning-Based Ultra-Wide-Field Fundus Imaging and True-Colour Confocal Scanning for Diagnosing Glaucoma
Source: J Clin Med. 2022 Jun 2;11(11):3168. doi: 10.3390/jcm11113168 (PMC9181263; doi:10.3390/jcm11113168)
Supplement: Supplementary file 1 [file jcm-11-03168-s001.zip › Table S1. CNN Accuracy.pdf]

Table S1. CNN Accuracy

Accuracy (%)	UWF fundus imaging using DL	True-color confocal scanner using DL
AlexNet	80.13	79.51
VGGNet	83.62	81.46
ResNet50	82.71	81.42

UWF, ultra-wide field; DL, deep learning; Comparison of representative Convolution Neural Network
