# Supplementary material for: Comparison between Deep-Learning-Based Ultra-Wide-Field Fundus Imaging and True-Colour Confocal Scanning for Diagnosing Glaucoma
Source: J Clin Med. 2022 Jun 2;11(11):3168. doi: 10.3390/jcm11113168 (PMC9181263; doi:10.3390/jcm11113168)
Supplement: Supplementary file 1 [file jcm-11-03168-s001.zip › Table S2. K-fold Cross Validation Verification(k=5).pdf]

Table S2. K-fold Cross Validation Verification (k=5)

Accuracy (%)	Test1	Test2	Test3	Test4	Test5	Mean	Accuracy
UWF fundus imaging using DL	87.14	80.64	81.41	84.61	88.41	84.44	83.62

UWF, ultra-wide field; DL, deep learning; five test groups and the accuracy evaluation and average of each group
